# Examining the Gastrointestinal and Immunomodulatory Effects of the Novel Probiotic *Bacillus subtilis* DE111

**DOI:** 10.3390/ijms22052453

**Published:** 2021-02-28

**Authors:** Kimberley E. Freedman, Jessica L. Hill, Yuren Wei, Allegra R. Vazquez, Diana S. Grubb, Roxanne E. Trotter, Scott D. Wrigley, Sarah A. Johnson, Michelle T. Foster, Tiffany L. Weir

**Affiliations:** 1 Intestinal Health Laboratory, Department of Food Science and Human Nutrition, Colorado State University, Fort Collins, CO 80523, USA; kim.freedman@colostate.edu (K.E.F.); yuren.wei@colostate.edu (Y.W.); allegra.stroud@colostate.edu (A.R.V.); diana.grubb@colostate.edu (D.S.G.); roxytrot@gmail.com (R.E.T.); wrigleys@rams.colostate.edu (S.D.W.); 2 Adipose Tissue Biology Laboratory, Department of Food Science and Human Nutrition, Colorado State University, Fort Collins, CO 80523, USA; Jessica.lynn.hill@colostate.edu (J.L.H.); michelle.foster@colostate.edu (M.T.F.); 3 Functional Foods & Human Health Laboratory, Department of Food Science and Human Nutrition, Colorado State University, Fort Collins, CO 80523, USA; sarah.johnson@colostate.edu

**Keywords:** *Bacillus subtilis*, flow cytometry, gastrointestinal health, immune cells, peripheral blood mononuclear cells (PBMC), probiotic, short chain fatty acid

## Abstract

Probiotics make up a large and growing segment of the commercial market of dietary supplements and are touted as offering a variety of human health benefits. Some of the purported positive impacts of probiotics include, but are not limited to, stabilization of the gut microbiota, prevention of gastrointestinal disorders and modulation of the host immune system. Current research suggests that the immunomodulatory effects of probiotics are strain-specific and vary in mode of action. Here, we examined the immunomodulatory properties of *Bacillus subtilis* strain DE111 in a healthy human population. In a pilot randomized, double blind, placebo-controlled four-week intervention, we examined peripheral blood mononuclear cells (PBMCs) at basal levels pre- and post-intervention, as well as in response to stimulation with bacterial lipopolysaccharide (LPS). We observed an increase in anti-inflammatory immune cell populations in response to ex vivo LPS stimulation of PBMCs in the DE111 intervention group. Overall perceived gastrointestinal health, microbiota, and circulating and fecal markers of inflammation (Il-6, sIgA) and gut barrier function (plasma zonulin) were largely unaffected by DE111 intervention, although the study may have been underpowered to detect these differences. These pilot data provide information and justification to conduct an appropriately powered clinical study to further examine the immunomodulatory potential of *B. subtilis* DE111 in human populations.

## 1. Introduction

The human GI tract houses roughly 3.8 × 10^13^ microorganisms comprising up to 1000 different species [1]. These microorganisms function to aid in digestion, protect the host from pathogens, and facilitate development of gut-associated lymphoid tissue (GALT) and other components of the immune system. Several studies have indicated that host–microbe interactions are required for proper GALT development [2,3,4]. Germ-free mouse models, mice lacking a gut microbiota, have been shown to have underdeveloped lymphoid organs as well as delayed and decreased somatic diversification of B cells [2]. In addition, they have reduced IgA production, decreased T regulatory cells, and impaired gut barrier function [3]. Colonization with commensal bacteria ameliorates these deleterious effects in germ-free mice and restores healthy immune function [4]. Studies of the interactions between the microbiota and immune cells reveal various immune-modulatory mechanisms, including activation of innate and humoral cell populations, induction of cytokine production, stimulation of secretory IgA production, competitive exclusion of pathogenic bacteria, and generation of bioactive metabolites, such as short chain fatty acids (SCFA) [5,6,7,8]. These interactions have led to the commercialization of probiotic organisms to promote immune health in consumers.

A novel probiotic bacterium of interest is *Bacillus subtilis* (*B. subtilis*), a rod-shaped, spore-forming, Gram-positive facultative aerobe [9,10]. In addition to its activity as a plant growth promoting organism found in the soil, *B. subtilis* is utilized for fermentation in Asian soy-based dishes such as natto and cheonggukjang. *Bacillus subtilis* has recently been considered for its probiotic potential because of its ability to form resistant spores, which enhance the shelf-life of these bacteria as well as their survival in the gastrointestinal (GI) tract. Additionally, recent evidence suggests that *B. subtilis* is part of the normal gut microbiota of humans [11] and may play a critical role in immune development [12] and promotion of GI health [9]. It completes its life cycle within the GI tract of humans and other vertebrates, as well as in invertebrate models [11,13,14]. In fact, GI isolates of *B. subtilis* have demonstrated increased sporulation compared to traditional lab-grown isolates of *B. subtilis* [13]. It has been hypothesized that, to combat the decreased efficiency of growth in the anaerobic conditions of the GI tract, *B. subtilis* creates an aerobic microenvironment via the production of exopolysaccharide (EPS) biofilms [14]. This EPS can also serve as a prebiotic, stimulating growth of other microbes in the GI tract and modulating immune cell populations that reduce intestinal inflammation in mouse models [15].

We investigated the safety and tolerability and potential immune modulatory effects of *B. subtilis* strain DE111 (DE111) in a healthy human population. We hypothesized that daily consumption of DE111 for four weeks would modulate the basal host immune status, as well as its response to a pro-inflammatory challenge, as determined via alteration in specific immune cell populations. We tested this hypothesis using a parallel arm, double-blind, randomized, placebo-controlled intervention study. Our outcome measures included examining the effects of *B. subtilis* on perceived gastrointestinal health, gut microbiota profiles, flow cytometric analysis of immune cell populations from cultured peripheral blood mononuclear cells (PBMCs), both with and without stimulation by bacterial lipopolysaccharide (LPS) and assessing circulating and fecal markers of inflammation and immune function. 

## 2. Results

### 2.1. Participant Characteristics and Study Compliance 

**Figure 1:**
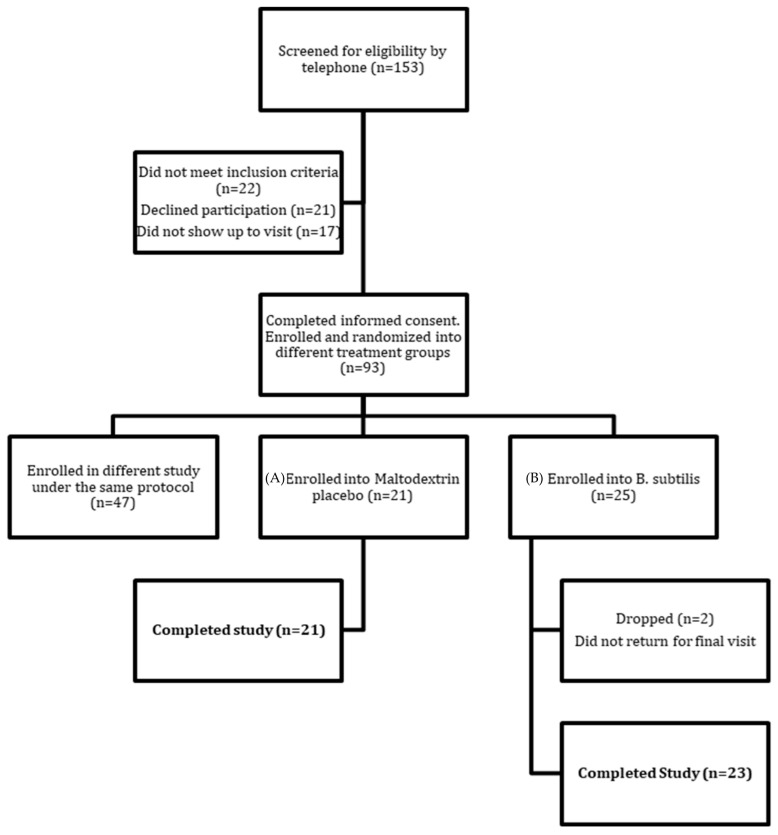
Consort flow diagram of study recruitment, randomization and completion.

Recruitment and enrollment were conducted under an umbrella protocol for two clinical studies. A total of 93 normal weight to mildly obese (BMI range: 20.0–34.9) healthy male and female adults between 20–62 years old were recruited to the protocol from an initial pool of 153 screened individuals (Figure 1). Of these 93 people, 46 individuals were enrolled into the current study and randomized to either DE111 (*n* = 25) or a placebo control group (*n* = 21). All 21 individuals enrolled in the placebo group completed the study and 23/25 people enrolled in the DE111 group completed the trial, with two individuals lost to follow-up, resulting in a total of 44 people completing the full study protocol.

Protocol compliance was measured as % of total pills consumed, based on the number of days on study minus the number of pills returned. Total study compliance with the intervention was ~96% with individual compliance ranging from 77 to 100%. Compliance to the specific interventions were as follows: Placebo: average compliance = 97% (range = 77–100%) and *B. subtilis* DE111: average compliance = 96% (range = 83–100%). Additionally, compliance with maintaining diet and body weight was achieved throughout the study. Neither intervention group nor diet influenced body weight over the course of the study. The average participant BMI was ~25 kg/m^2^, regardless of intervention group, and the average weight fluctuated by <1 kg. There were no differences between groups in blood glucose or other metabolic parameters and no effect of treatment over time (Supplemental Figure S1). Baseline characteristics for participants included in this study are presented in Table 1.

### 2.2. Dietary Recall

There was poor compliance for completing the self-reported 24 h food recalls, with only ~54% of participants providing both baseline and four-week dietary intake information. Compliance within each of the intervention groups was as follows: Placebo: average compliance = 48% and *B. subtilis* DE111: average compliance = 59%. Despite poor reporting compliance, the available data showed no significant differences in dietary intake of total calories or the various nutrient classes between baseline and study completion within either group (Table 2). There were also no significant differences between groups regarding change in intake over the course of the study.

### 2.3. Adverse Events

A total of six participants in the *B. subtilis* DE111 group reported adverse events, with the severity of events reported as moderate or unspecified. Adverse events included constipation, flatulence, fatigue, anxiety, and diarrhea. Two of these reports were not attributed to study participation. One participant experiencing diarrhea reported two days of watery stool. The duration of the other adverse events was not reported, but included bloating and flatulence. One participant on the maltodextrin placebo reported having flatulence and a lack of concentration, not attributed to the study. No adverse events were reported as severe or life-threatening, and all participants reporting adverse events remained in the study.

### 2.4. Perceived Gastrointestinal Health

**Figure 2:**
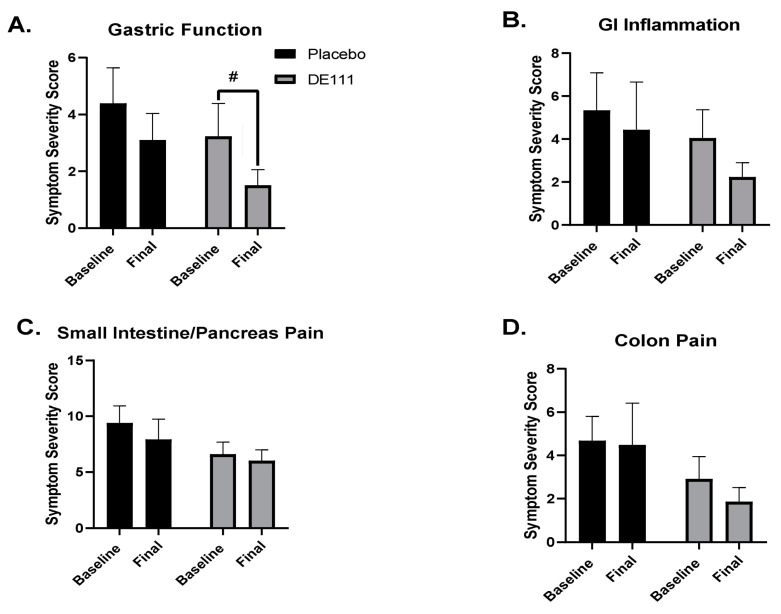
Symptom severity scores were based on answers to a GI symptom questionnaire by Metagenics [16] that is designed to provide a functional measure to assess symptoms associated with gastric function (**A**), gastrointestinal inflammation (**B**), small intestine/pancreas pain (**C**), and colon pain (**D**). Error bars represent ±SEM, # indicates a statistical trend (*p* = 0.051–0.08).

Although more participants in the intervention group reported adverse events compared to the placebo group, there were no negative changes in self-reported measures of gastrointestinal health. In fact, there was a significant overall effect for time in symptom severity scores related to gastric function (Figure 2A; *p* = 0.008; CI = 0.41, 2.61), suggesting overall improvement in the study population, regardless of intervention group. Although the main effect was driven by decreases in symptom severity scores in both the placebo and DE111 groups, only the DE111 group showed a trending (defined in this study as *p* = 0.51 to 0.08) reduction in severity score from baseline to post-intervention (*p* = 0.056; CI= −3.49, 0.04). Gastrointestinal inflammation, small intestine and pancreas pain, and colon pain scores did not change over time with the probiotic intervention or placebo, nor were there any significant differences between the groups (Figure 2).

### 2.5. Quantitative PCR (qPCR) and 16s Microbiota Analysis

**Figure 3:**
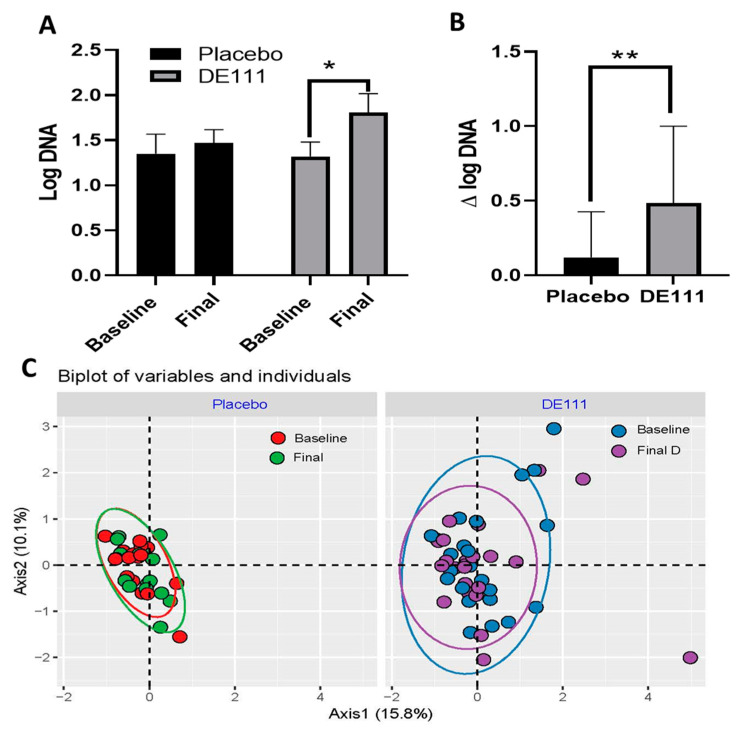
Levels of *B. subtilis* DNA detected in fecal samples collected pre- and post-intervention (**A**) and the change in logDNA from baseline between the two intervention groups (**B**) indicate that DE111 consumption increases *B. subtilis* in the GI tract. However, four weeks of DE111 consumption did not significantly alter the composition and structure of the gut microbiota (**C**). Error bars represent ±SEM. * indicates *p*-values <0.05 and ** indicate *p*-values <0.01. Ellipses in the PCoA represent the 95% CI.

Using qPCR, our 2-way mixed effects model revealed a significant effect for time in *B. subtilis* group log DNA detected in stool (*p* = 0.049; CI= −0.61, 0.00). Post-hoc Sidak tests identified that this was driven by an increase in *B. subtilis* group DNA in stool samples from individuals in the intervention group (Figure 3A; *p* = 0.044; CI = 0.01, 0.96). There were no significant differences between timepoints in the placebo group or between the two interventions. Although the amount of *B. subtilis* group DNA was not significantly different between the final timepoint for the probiotic intervention and control (*p* = 0.35), the baseline adjusted difference in *B. subtilis* DNA between the two groups was significant using an unpaired t-test (Figure 3B; *p* = 0.01; CI = −0.24, 0.97). Interestingly, *B. subtilis* group DNA was detected in most samples, supporting previous reports that these organisms are naturally present in the human gut [11]. However, it is also important to note that in mixed microbial communities, the primers used will also replicate DNA from other species in the *Bacillus subtilis* group (i.e., *B. atrophaeus, B. licheniformis, B. amyloliquefaciens* and *B. pumilus*), suggesting that some of the baseline detection may be due to the presence of these closely related organisms. 

Using 16s rRNA sequencing, we next sought to determine whether DE111 consumption altered the community structure of the gut microbiome. We did not detect any significant differences in observed or estimated microbial richness (Supplemental Table S1) or in Bray–Curtis distances between intervention groups or within a given intervention over time (Figure 3C). 

### 2.6. Short Chain Fatty Acid Analysis

**Figure 4:**
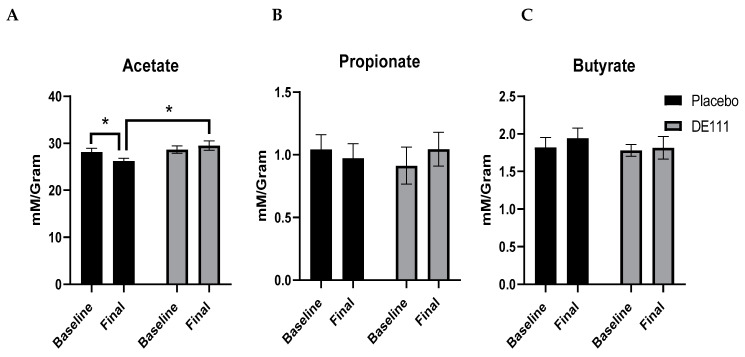
The major microbially-produced short chain fatty acids (SCFA), acetate (**A**), propionate (**B**), and butyrate (**C**) quantified in feces at baseline and post-intervention. Error bars represent ±SEM, *denotes *p* < 0.05.

Using gas chromatography, we measured fecal short chain fatty acids (SCFAs) at both the start and completion of the intervention period. On average, fecal samples contained about 30 mM/gram of acetate, and only between 1–2 mM/gram of propionate and butyrate. There was a significant interaction effect for acetate (time x treatment; *p* = 0.047; CI = 0.04, 4.99), as well as a trending main effect for intervention (*p* = 0.062; CI= −3.62, 0.10). This was primarily driven by a significant decrease in acetate in the placebo group over the four-week intervention (*p* = 0.046; CI= −3.78, −0.03), resulting in significantly higher levels of acetate in the DE111 compared to the placebo post-intervention (Figure 4A; *p* = 0.017; CI = 0.47, 5.57). There were no significant effects either with probiotic intervention, or over time, for propionate or butyrate (Figure 4B,C). 

### 2.7. Immune Cell Populations

#### 2.7.1. Basal Cell Counts

**Figure 5:**
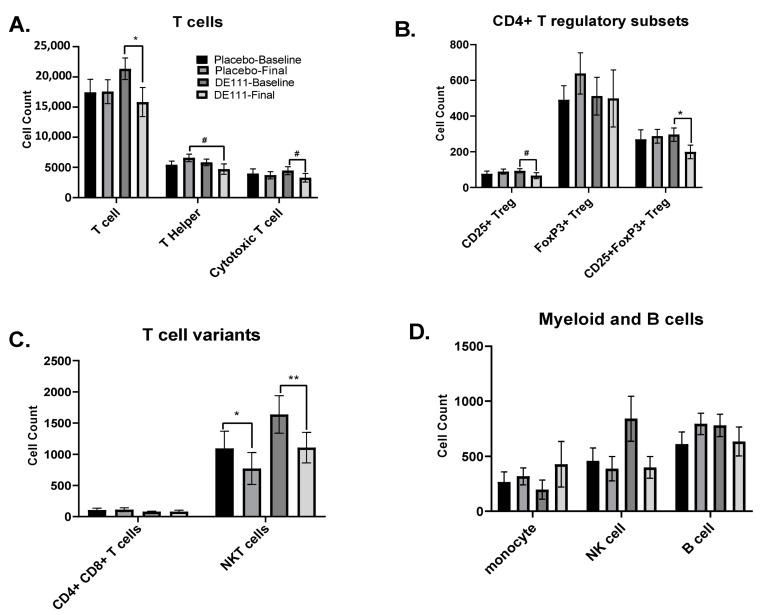
Basal immune status at baseline and post-intervention in Placebo and DE111 groups: Total CD3+ T cell populations (**A**), CD4+ T regulatory subpopulations (**B**), T cell variants (**C**), and myeloid and B cells (**D**). Error bars represent ± SEM. * denotes *p* < 0.05, **denotes *p* < 0.01, and # denotes a *p*-value between 0.05–0.08.

Collected PBMCs were cultured and assessed by flow cytometry to determine immune cell populations. There was a significant effect of DE111 intervention over time resulting in significant decreases in CD3+ T cells (Figure 5A; *p* = 0.02; CI = −12,111, 1066) and CD25+FoxP3+ T regulatory cells (Figure 5B; *p* = 0.03; CI = −234, 40) when comparing the baseline visit to the final visit. There was also a trending reduction in the DE111 group over time in CD8+ cytotoxic T cells (*p* = 0.07; CI = −3291, 953) and CD25+ T regulatory cells (*p* = 0.06; CI = −75, 21; Figure 5A,B). There was also a trending effect of DE111 intervention resulting in decreased CD4+ T helper cells post-intervention in the DE111 group compared to placebo (*p* = 0.06; CI = −5094, 1378). Both the placebo and DE111 groups showed significant decreases in NKT cells between the baseline and final visits (Figure 5C; *p* = 0.04; CI = −885, 244 and *p* = 0.01; CI = −1084, 25, respectively). There were no significant differences either between or within groups for myeloid cells or B cells (Figure 5D). 

#### 2.7.2. LPS-Stimulated Cell Counts

**Figure 6:**
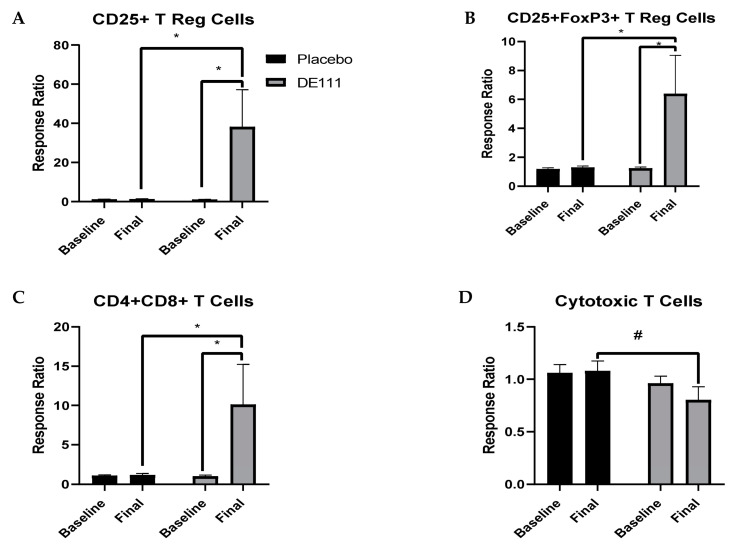
Cellular responses to LPS stimulation, calculated as stimulated/basal cell counts for CD25+ (**A**) and CD25+FoxP3+ (**B**) regulatory T cell populations, as well as double-positive CD4+CD8+ T cells (**C**) and CD8+ cytotoxic T cells (**D**). Error bars represent ± SEM. * denotes *p* < 0.05 and # denotes a *p*-value between 0.05–0.08.

In order to better understand how DE111 intervention impacted immune cell responses, we stimulated cultured PBMCs with bacterial lipopolysaccharide (LPS). The immune cell response was calculated as a ratio of LPS-stimulated cell counts/basal cell counts. In general, most of the cell counts were not significantly altered with LPS-stimulation (i.e., response ratio of ~1; data not shown). However, CD25+ and CD25+FoxP3+ T regulatory cell counts were significantly increased in the DE111 group, both from baseline levels and at the final timepoint between the two groups (Figure 6A,B; Table 3). Similarly, DE111 intervention led to a significant increase in CD4+CD8+ double-positive T cells both within and between groups (Figure 6C; Table 3). In contrast, CD8+ cytotoxic T cells were reduced after DE111 intervention compared to the placebo group (Figure 6D, Table 3). There were no significant differences in the percent proliferating cells after LPS stimulation (Supplemental Figure S2).

### 2.8. Inflammation and Gut Barrier Function

We assayed circulating levels of IL-6 and plasma zonulin to determine basal inflammation and gut permeability before and after the intervention. There were no significant differences either between or within groups for any of these parameters. Finally, we also examined sIgA in the stool to assess intestinal immune responses. Again, we observed no differences between groups or within an intervention over time for these parameters. (Supplemental Figure S3).

## 3. Discussion

Spore-forming *Bacillus subtilis* is becoming increasingly popular as a probiotic supplement due to its enhanced shelf life and survivability in the human digestive tract relative to other bacterial species. Although the in vivo viability, safety, and tolerability of several strains of *B. subtilis* have been assessed in appropriate human populations [17,18,19,20], little is known about other specific benefits these bacteria may convey. We recently demonstrated that four weeks of *B. subtilis* DE111 supplementation was associated with improved lipid parameters and endothelial function in healthy adults [21]. In addition, several in vitro and animal model studies have suggested that *B. subtilis* may modulate the mucosal immune system via production of antimicrobial peptides, exopolysaccharides, and quorum-sensing molecules, as well as through favorable modifications to the gut microbiota [22,23,24]. In elderly patients, intermittent use of *B. subtilis* strain CU1 increased fecal and salivary sIgA [25]. Beyond this, the effects of *B. subtilis* on the human immune system are still largely unexplored, and to our knowledge have not been assessed in an LPS-challenge model. 

Here, we observed that oral consumption of *B. subtilis* DE111 for four weeks induced changes in peripheral immune cell populations. We interpret these results as potentially due to reduced systemic inflammation after probiotic intervention, although limited compliance in dietary intake reporting limit our ability to directly attribute these effects to the probiotic. Specifically, we observed a DE111-associated decrease in several T cell subsets, driven by modest reductions in CD4+ helper T cells and CD8+ cytotoxic T cells, and more substantially by decreases in CD25+ and CD25+FoxP3+ Tregs. There have been a few similar studies looking at T cell changes in human PBMCs either after oral probiotic feeding or co-culture of the PBMCs with cell free bacterial supernatants. For example, in contrast to our results, FoxP3+ Tregs were increased after eight weeks of consuming *Bifidobacterium infantis* [26]. In contrast, co-culture of cell free supernatants of *Lactobacillus reuteri* with *Staphylococcus aureus*-stimulated PBMCs had no effect on FoxP3+ Tregs and dampened activation and proliferation of both CD4+ helper T cells and CD8+ cytotoxic T cells [27]. Although not directly relevant to our experimental model, there have been several studies where PBMCs or isolated cell populations were directly co-cultured with various probiotics, and these results suggest that stimulation or suppression of specific cell populations or their secreted cytokines are highly variable depending on probiotic species tested and exposure times [28,29]. In the context of these existing literature, our results support the broader hypothesis that probiotic effects on immune function are species- or strain-specific and highlight the need for development of standardized assays to assess immune function within human populations. 

It is also important to note that most immune cells found in peripheral circulation are naïve lymphocytes and myeloid cells. When myeloid cells activate naïve lymphocytes via antigen presentation and cytokine stimulation, the lymphocytes migrate to the lymph nodes as effector cells. Our observed decreases in these T cell subsets, therefore, could reflect an anti-inflammatory effect of DE111 via decreased cell activation resulting in decreased overall circulating T cells. However, without measurement of specific myeloid and T cell activation markers, respectively, these hypotheses are based on speculation. 

In addition to assaying basal cell counts before and after probiotic intervention, we also sought to determine how DE111 impacted the immune response to a pro-inflammatory challenge. To test this, we cultured PBMCs with bacterial lipopolysaccharide. We chose to use a chronic (low-dose LPS for 72 h) rather than acute (high-dose LPS for shorter duration) stimulation model because several recent studies have indicated that environmental factors such as poor diet and excess body weight lead to a state referred to as “metabolic endotoxemia”, which over time can result in development of several chronic diseases in an otherwise healthy population [30]. In the context of this model, we observed DE111-associated increases in CD25+ and CD25+FoxP3+ Tregs, as well as double-positive CD4+CD8+ T cells after LPS stimulation. These regulatory T cell subsets act to resolve inflammation and modulate immune responses. While the function of the double-positive CD4+CD8+ T cells is still unclear, they are surmised to play roles in both suppressive and cytotoxic responses [31] and exhibit an increased capacity to produce cytokines and display phenotypic profiles associated with memory T lymphocytes [32]. Interestingly, there was a slight decrease in CD8+ cytotoxic T cells. This may be due to the fact that these cells primarily respond to viral pathogens and we challenged PBMCs with a bacterial effector molecule or this could be the result of transcriptional reprograming towards a CD4+CD8+ phenotype [31]. Overall, our results indicate an expansion of regulatory immune cells from DE111-treated individuals in response to low-grade, pro-inflammatory stimulation. Therefore, DE111 may be useful in suppressing responses driven by obesity and diet-derived metabolic endotoxemia. This is consistent with our previous observation of improved lipid parameters and endothelial function in DE111-treated individuals, which can result from metabolic dysregulation driven by inflammation [21]. Finally, it should be noted that even within the DE111 group, the responses between individuals were highly variable and the observed differences in immune cell populations was largely driven by a group of responders. Therefore, further study into the factors that drive individual responsiveness to DE111 in an appropriately powered intervention study is also warranted.

One limitation to establishing the immunomodulatory effects of *B. subtilis* DE111 in our study is the under-representation of outcomes related to mucosal immune activity. Probiotics largely exert their immunomodulatory effects through direct interactions with immune cells in the intestinal mucosa. The mucosal immune system hosts the vast majority of innate and effector immune cells as well as specialized cells that limit systemic inflammation by acting locally via an array of anti-inflammatory mechanisms [33]. However, due to the limitations of obtaining mucosal tissue from healthy human participants, studying PBMCs in vitro is a commonly used proxy, particularly when monitoring immune responses to enteric pathogens [34], although it may not accurately reflect DE111′s effects on mucosal immunity. To more thoroughly assess mucosal immune responses and gut barrier function, we measured fecal sIgA and plasma zonulin. We also assessed plasma IL-6 levels as a circulating inflammatory marker. However, none of these measures were affected by our current probiotic intervention protocol. These results are consistent with a study in Division 1 athletes who also consumed *B. subtilis* DE111 at the same dose for 12 weeks and reported no changes in plasma zonulin [18]. Unlike the current study, they did report a decrease in a circulating inflammatory molecule (TNF-a) and an increase in sIgA in the DE111-treated group, although this was measured in saliva rather than in feces. These differences may have resulted from the increased duration of probiotic use or because the athletes were all involved in an intense physical training program during the study that likely resulted in elevated basal inflammation. In contrast to these results, Lefevre et al. suggest that intermittent use of *B. subtilis* CU1 did not alter circulating inflammatory markers compared to placebo, although they did report a transient increase in IFN-γ from baseline levels after the first cycle of probiotics [25]. They also reported elevated fecal and salivary sIgA with probiotic intervention relative to the placebo [25]. In a murine model, oral administration of *B. subtilis* spores also increased serum IFN-γ as well as splenic macrophage and natural killer cell activity [35]. While we did not measure IFN-γ, this may be an important marker to measure in future studies. In general, an understanding of the background inflammatory environment in which these cell populations and cytokines are measured will be key in interpreting immunologic responses to probiotics. 

The results from our secondary outcome measures, specifically safety and tolerability of *B. subtilis* DE111 intake, as well as its overall impact on microbiota stability, were largely confirmatory. For example, a study that administered five million CFU/day of *B. subtilis* DE111 to 41 healthy adult participants reported after ~20 days of consumption that blood metabolic parameters remained within normal ranges and there were no significant gastrointestinal disturbances or alterations to bowel movements [20]. In addition, their reports of increased fecal counts of *B. subtilis* are consistent with the DE111-associated increases that we report in our qPCR data and support that these bacteria do survive intestinal transit [14]. A recently published study examined the impact of *B. subtilis* DE111 on the gut microbiota of preschool aged children after an 8-week consumption period [19]. They concluded that DE111 did not disrupt the global microbiota community structure, as evidenced by a lack of differences pre- and post-intervention in beta-diversity (determined using Bray–Curtis dissimilarity), which is consistent with our findings. They also did not see pre- and post-intervention changes in taxa richness, although diversity did increase with DE111 consumption. While we did not see concurrent changes in bacterial diversity, this may be due to the differences in participant age of the study populations. Their study focused on children, who have a much more dynamic microbiota than adults.

In terms of whether DE111 consumption impacts microbial metabolism and fermentative capacity of the microbiome, we are the first to report fecal SCFA concentrations in humans. We saw no changes in SCFA that could be attributed to the study intervention, and a small but significant decrease in acetate in the control group, which cannot be explained by the available data, but may be due to diet changes that were not captured since dietary reporting compliance was low. One study in dogs fed a *B. subtilis* supplement did report a slight increase in propionate, but no change in concentrations of butyrate or acetate [36], and other studies have demonstrated that exopolysaccharides, such as those produced by *Bacillus*, can be fermented by gut bacteria to enhance SCFA production [37]. Therefore, it would be important to monitor this parameter in studies using other doses or durations of *Bacillus*-based probiotics. Finally, it is important to note that we had increased numbers of adverse events in the probiotic intervention group compared to the placebo control group. However, none of these events were severe enough to result in study attrition. Furthermore, there was a general lack of difference in perceived GI symptom severity between the placebo and intervention group over the intervention period, which, when taken in context of the existing literature on DE111 use, suggest that this probiotic is safe, tolerable, and does not disrupt the general homeostasis of the gut microbiota.

A limitation of the current study was the use of cryopreserved PBMCs for assessing immune cell populations. Freshly isolated cells have been shown to respond differently to LPS stimulation than cells that have been frozen and thawed prior to culturing and experimentation [38,39]. However, due to the duration of the study and the need to minimize variability during staining and flow cytometric analysis, it was most feasible to freeze cells and process them as large cohorts after completion of the clinical trial. Despite this limitation, our study demonstrated that consumption of *B. subtilis* DE111 displayed immunomodulatory and anti-inflammatory effects on several T cell subsets. This is supported by the observed reduction in immune cell populations within the basal state, along with expansion of regulatory immune cells in response to LPS challenge. To further elucidate *B. subtilis* DE111 interactions and mode of immune modulation within the gut, direct stimulation of immune cells in mucosal tissue should be explored. These findings show preliminary yet promising results for utilizing DE111 as an immune-modulating probiotic supplement in a healthy adult population.

## 4. Materials and Methods 

### 4.1. Study Design

This study was conducted as a parallel-arm, randomized, double-blind, placebo-controlled, four-week intervention trial in healthy adults. Participant eligibility was determined by telephone interview, and subjects arrived at the Food and Nutrition Clinical Research Laboratory (FNCRL) at Colorado State University, to provide written consent. Eligibility was confirmed through anthropometric measurements of height and weight to determine BMI. After BMI was confirmed, participants were randomly assigned to 1 of 2 coded interventions of maltodextrin placebo (*n* = 21) or *Bacillus subtilis* strain DE111 spores (1 × 10^9^ CFU; Deerland Probiotics & Enzymes, Kennesaw, GA, USA) (*n* = 25). Over the course of the 4-week intervention, participants were asked to consume one 15 mg capsule per day containing the dispensed treatment. Compliance with study protocols was determined for both capsule consumption and diet assessment. Compliance with capsule consumption was calculated for each individual as percent capsules consumed (capsules given - capsules returned)/total days on study and then averaged across the intervention group. Dietary reporting compliance was calculated as percent of total participants per group returning all four dietary assessment records.

### 4.2. Participant Recruitment

Male and female adults aged 20–65 years of age with a BMI of 20−34.9 kg/m^2^ were eligible for recruitment into the study. Recruitment occurred in the Fort Collins, CO area by means of social media, email and posted fliers. Respondents were pre-screened for eligibility via telephone. Eligible respondents were scheduled for a clinical screening to further confirm eligibility and obtain consent. Exclusion criteria included the following: (A) those under 18 or over 65 years of age; (B) BMI outside the range of 20–34.9 kg/m^2^); (C) pregnancy or breastfeeding; (C) current prescription medication use or antibiotic use in the previous 2 months; (D) diagnosis of gastrointestinal cancers or diseases such as malabsorption, celiac, liver or kidney disease or other conditions known to affect the microbiota or nutrient absorption. Enrolled participants were instructed to maintain normal diet and exercise patterns and abstain from use of supplemental pre- or probiotics, and to limit alcohol consumption to one drink per day for women and two drinks per day for men, or no more than seven drinks per week. Participants were randomized and enrolled into different treatment groups, forty-seven of which were enrolled into a separate study under the same protocol (Protocol #19-9145H, ClinicalTrials.org NCT04511221). Of the total enrolled participants (*n* = 94), 44 completed the currently outlined study. 

Participants were asked to fast for eight hours (including no caffeine, soda, tea, etc.) and abstain from exercise for 12 h prior to arrival in the clinic. Additionally, participants were asked to delay consumption of any medication or dietary supplements for 24 h prior to the study visit. Participants arrived at the FNCRL for their baseline visit to undergo sample collections (blood and stool) and analysis procedures (weight/height, blood pressure, pulse wave and endothelial function analysis, gastrointestinal symptom screening, and medical health history questionnaire), as previously described [21]. Whole blood was collected from the antecubital vein and analyzed for metabolic and lipid parameters using a Piccolo Xpress (Abaxis; Union City, CA, USA) with Piccolo Metlyte plus CRP and Lipid Panel reagent discs. The methods and results for blood pressure and endothelial function are published elsewhere [21]. Stool samples were collected by participants in study-provided collection containers and stored under refrigeration prior to being returned to the clinic. Aliquots were removed for DNA and short chain fatty acid (SCFA) extraction and stored at −80 °C until processed. 

Over the duration of the intervention period, participants were asked to maintain their regular diet and exercise regime, and record bowel movements in a daily log using a Bristol stool chart. In addition, they were asked to complete two 24 hr dietary recalls (1 weekday and 1 weekend day), prior to each of their clinic visits, via computer software Automated Self-Administered 24 h dietary assessment tool (ASA24) developed by the National Institutes of Health, National Cancer Institute. At the end of the 4-week intervention period, these analyses and sample collections were conducted again at the final visit. Participants were asked to return any unused capsules to establish intervention compliance. All participants provided informed written consent prior to participating in the study, which was conducted in accordance with the Declaration of Helsinki and approved by the Colorado State University Institutional Review Board for Human Subjects (Protocol #19-9145H). 

### 4.3. Isolation of Peripheral Blood Mononuclear Cells

Ten milliliters of whole blood was collected from antecubital veins into ethylenediaminetetraacetic acid (EDTA)-treated vacutainer tubes. PBMCs were isolated from whole blood within six hours of collection via density gradient centrifugation and a series of washes. Initially, whole blood was diluted with 1X Phosphate Buffer Solution (PBS) + 2% Fetal Bovine Serum (FBS) (Atlas Biologics; Fort Collins, CO) at a 1:1 ratio and transferred into 50 mL SepMate^TM^ tubes containing 17 mL of density gradient medium, Lymphoprep (Stemcell Technologies Inc; Vancouver, Canada). Tubes were centrifuged for 10 min at 1200× *g* at room temperature. The separated plasma and PBMCs were poured off and diluted with an equivalent volume of 1× PBS + 2% FBS and centrifuged at 300× *g* for 8 min. Tubes were decanted and pelleted cells were resuspended in an equivalent volume of 1× PBS + 2% FBS for a final wash and centrifugation. Cells were counted using a Cellometer Auto T4 Cell Counter (Nexcelom Bioscience LLC; Lawrence, MA, USA) to calculate the appropriate volume of cell freezing media, CryoStor (Biolife Solutions; Bothwell, WA, USA). Pelleted cells were re-suspended in CryoStor and placed in Mr. Frosty Containers at −80 °C for 12–24 h before final storage in liquid nitrogen.

### 4.4. PBMC Culture and Stimulation

All cell processing was performed aseptically in a laminar flow hood. A complete culture medium containing 1× RPMI-1640 (Corning; Corning, NY, USA), 10% FBS (Atlas Biologics; Fort Collins, CO, USA), and 1% penicillin/streptomycin [100 U/mL penicillin and 100 μg/mL streptomycin] (HyClone; Tianjin, China) was warmed to 37 °C in a water bath. Frozen human PBMCs were rapidly thawed in a 37 °C water bath. Samples were then transferred to a 15 mL conical tube and warm media was added slowly at a rate of 1 mL/5 sec to a final volume of 10 mL. Cells were then centrifuged for 8 min at 300× *g* at 25 °C and decanted, followed by two additional washes. Finally, cells were plated for an overnight recovery period at about 1–2 × 10^6^ cells/mL.

After the 24 h rest, cells were counted and seeded at a desired concentration between 2–5 × 10^5^ cells/well in a U-bottom, 96-well untreated plate to prevent cell adherence. All samples were plated in duplicate. Appropriate wells were then labeled with proliferation marker, CFSE (Biolegend; San Diego, CA, USA) according to the manufacturer’s instructions for a final concentration of 1 μM. Finally, designated wells were spiked with *E. coli* LPS (Sigma-Aldrich; St. Louis, MO, USA) to achieve a final concentration of 1 μg/mL. Cells were incubated at 37 °C and 5% CO_2_ for 72 h. Post incubation, sample plates were centrifuged for 5 min at 400× *g* at 4 °C and pelleted cells were stained for flow cytometry.

### 4.5. Flow Cytometric Analysis

PBMCs were washed with 1X PBS, centrifuged (500 g, 4 min, 4 °C), resuspended in LIVE/DEAD fixable Yellow (Invitrogen, Carlsbad, CA, USA) and incubated on ice for 30 min. Cells were then pelleted, washed, resuspended in Human TruStain FcX receptor blocking solution (Biolegend, San Diego, CA, USA) and incubated on ice for 10 min. After blocking, cells were surface stained with the following fluorescently conjugated antibodies: APC-anti-CD25 (clone M-A251), BV570-anti-CD3 (clone UCHT1), Pac Blue-anti-CD4 (clone SK3), BV750-anti-CD8 (clone SK1), BV605-anti-CD19 (clone HIB19), AF700-anti-CD14 (clone HCD14), BV785-anti-CD16 (clone 3G8), PE-anti-CD56 (clone 5.1H11), and incubated on ice for 30 min. Super Bright Complete Staining Buffer (eBioscience, San Diego, CA, USA) was used per the manufactures instructions in stain master mixes which contained more than one polymer dye. Cells were then processed for nuclear staining using the Foxp3/Transcription Factor Staining Buffer Set (eBioscience) according to manufactures instructions and stained with BV421-anti-FoxP3 (clone 206D). All antibodies were purchased from Biolegend (Biolegend; San Diego, CA, USA). Samples were analyzed using a Cytek Biosciences Aurora 4 laser spectral flow cytometer (Cytek Biosciences; Fremont, CA, USA and acquired data were analyzed using FlowJo™ ver. 10.6.1 Software (Becton Dickinson, Franklin Lakes, NJ, USA).

### 4.6. ELISA Assays

Plasma collected from whole blood was used to measure circulating IL-6 (Invitrogen, Carlsbad, CA, USA) and zonulin (Elabscience, Houston, TX, USA), and fecal samples were assayed for secretory IgA (BioVendor LLC, Asheville, NC, USA) by ELISA. All kits were run according to manufacturer’s instructions. Target analytes were quantified by fitting to standard curves provided with each kit. 

### 4.7. Fecal DNA Extraction and Analysis

Fecal DNA was extracted from samples using the FastDNA Extraction Kit (MP Biomedical, Irvine, CA, USA) and used for qPCR and preparation of 16s sequencing libraries. Prior to amplification all sample DNA was diluted to ~10 ng/µL and 1 µL of DNA was added to each PCR reaction. Reactions for each sample were run in duplicate. Quantitative PCR was conducted with *B. subtilis* group-specific primers ([40]; Forward: 5′-AAGTCGAGCGGACAGATGG-3′; Reverse: 5′-CCAGTTTCCAATGACCCTCCCC-3′) on a CFX96 Touch Thermocycler (Bio-Rad Laboratories, Hercules, CA, USA) using the following amplification program: 95 °C for 3 min followed by 30 cycles of 95 °C for 15 s, 60 °C for 30 s, 72 °C for 30 s with a final extension 72 °C for 5 min. *Bacillus subtilis* levels in samples was quantified by generation of standard curves using purified *B. subtilis* DNA (dilutions: 2–2 × 10^−5^ ng DNA/rxn). Sequencing libraries were prepared following the Earth Microbiome Project protocols, as previously described [16]. Amplicon libraries were sequenced on an Illumina Miseq at the Next Generation Sequencing Core at Colorado State University. All sequence data were processed using the DADA2 pipeline in QIIME 2 ver. 2020.8.0. Alpha diversity parameters were calculated in QIIME 2 and MyPhyloDB ver. 1.2.1 was used for calculating Bray–Curtis distances and PCoA visualizations.

### 4.8. SCFA Extraction and Analysis

SCFA acids were extracted from fecal samples and quantified by Gas Chromatograph with Flame Ionization Detection (GC-FID) as previously described [41]. Briefly, one gram of feces was sonicated in acidified, HPLC-grade water (pH brought to 2.5 with HCl) and allowed to sit at RT for 10 min. Samples were then centrifuged, and supernatants removed and frozen overnight at −80 °C. The supernatants were then thawed, centrifuged again to sediment any remaining particulate matter, and filtered through 0.45-micron filters. Collected supernatant was analyzed on a GC-FID (Agilent 6890 Plus GC Series, Agilent 7683 Injector Series, GC Column: TG-WAXMS A 30 m × 0.25 mm × 0.25 μm). Quantities of specific SCFA were determined through generation of standard curves of purified commercial chemicals.

### 4.9. Statistical Analysis

R software version 3.6.1 and Graphpad Prism version 8.3.0 were used for all statistical analyses. Linear mixed effects model (time x treatment) with post hoc Sidak’s multiple comparison test was used for all analyses. Clinical data were evaluated for assumption of normality and equal variance. Incomplete sample sets with missing baseline and/or final blood samples were excluded from the immune cell counts (analyzed: *n* = 17 for placebo group, *n* = 18 for DE111), but were included in all other analyses. Microbiome alpha-diversity comparisons were done using non-parametric Kruskal–Wallis tests and Bray–Curtis distances were evaluated for significance by PERMANOVA with 1000 iterations. 

## Figures and Tables

**Table 1:** Participant Baseline Characteristics.

Intervention	Male	Female	Height (cm)	Weight (kg)	BMI (kg/m^2^)	Age (yr)
Placebo (*n* = 21)	8	13	169.4 ± 7.3	71.0 ± 10.0	24.7 ± 2.8	34.4 ± 13.0
*Bacillus subtilis* DE111 (*n* = 25)	10	15	170.8 ± 9.1	72.6 ± 9.3	24.9 ± 2.4	36.9 ± 12.9

Data represents mean ± SD.

**Table 2:** Self-Reported Diet Intake Based on Two 24-Hour Recalls.

	Placebo (*n* = 10)		DE111 (*n* = 13)	
	Baseline	Final	Baseline	Final
**Energy (kcal)**	2269 ± 999	2089 ± 982	2238 ± 741	2389 ± 638
**PRO (g)**	99 ± 50	96 ± 50	96 ± 40	102 ± 48
**TFAT (g)**	103 ± 56	86 ± 42	95 ± 40	101 ± 32
**SFAT(g)**	33 ± 18	27 ± 14	32 ± 19	34 ± 15
**CHOL (mg)**	358 ± 259	324 ± 271	367 ± 221	439 ± 308
**CARB (g)**	240 ± 104	225 ± 118	223 ± 79	237 ± 73
**FIBER (g)**	25 ± 11	22 ± 12	21 ± 6	27 ± 10

Data represents mean ± SD. Abbreviations: PRO (protein), TFAT (total fat), SFAT (saturated fat), CHOL (cholesterol), CARB (carbohydrates). No significant differences were observed within or between intervention groups.

**Table 3:** Statistical analysis (Mixed-Effects Model with Sidak post hoc comparisons) for LPS-induced response ratios of significant and trending changes in immune cell populations.

Main Effects	Time		Treatment		Interaction	
Cell Type	*p*-value	95% CI Difference	*p*-value	95% CI Difference	*p*-value	95% CI (A1-B1)-(A2-B2)
CD25+	0.070	−38.80, 1.61	0.073	−38.69,1.80	0.070	−3.27, 77.54
CD25+FoxP3+	0.068	−5.47, 0.21	0.074	−5.42, 0.26	0.080	−0.63, 10.73
CD4+CD8+	0.079	−9.71, 0.55	0.090	−9.55, 0.71	0.084	−1.25, 19.26
CD8+ Cytotoxic	0.435	−0.11, 0.26	0.064	−0.01, 0.39	0.334	−0.55, 0.19
**Sidak Post hoc**	**DE111 (Baseline-Final)**			**DE111 vs Placebo (Final)**		
**Cell Type**	***p*-value**	**95% CI** **Difference**		***p*-value**	**95% CI** **Difference**	
CD25+	0.021	4.99, 69.34		0.022	4.59, 69.43	
CD25+FoxP3+	0.023	0.63, 9.68		0.025	0.55, 9.66	
CD4+CD8+	0.027	0.90, 17.28		0.036	0.49, 17.36	
CD8+ Cytotoxic	0.380	−0.46, 0.14		0.088	−0.59, 0.03	

## Data Availability

16s sequencing data and associated metadata are publicly available under the project name PHAGE2 at https://myphylodb.azurecloudgov.us/myPhyloDB/home/.

## Supplementary Materials

Supplementary materials can be found at https://www.mdpi.com/1422-0067/22/5/2453/s1, Table S1: Alpha Diversity Measures, Figure S1: Metabolic Parameters, Figure S2: Percent Proliferating Cells, Figure S3: Inflammatory Markers.

## Abbreviations

CFU	Colony forming units
GI	Gastrointestinal
FNCRL	Food and Nutrition Clinical Research Laboratory
PBMC	Peripheral blood mononuclear cell
SCFA	Short chain fatty acid
