# Supplementary material for: Examining the Gastrointestinal and Immunomodulatory Effects of the Novel Probiotic Bacillus subtilis DE111
Source: Int J Mol Sci. 2021 Feb 28;22(5):2453. doi: 10.3390/ijms22052453 (PMC7957723; doi:10.3390/ijms22052453)

International Journal of *Molecular Sciences*

MDPI

	Placebo (n = 16)		DE111 (n = 23)	
	Baseline	Final	Baseline	Final
**Pileu's Evenness**	0.70 ± 0.05	0.71 ± 0.04	0.68 ± 0.09	0.69 ± 0.08
**Faith's Phylogenetic Diversity**	11.15 ± 2.36	11.41 ± 1.98	11.07 ± 2.65	11.26 ± 2.50
**Shannon's Diversity**	5.10 ± 0.50	5.22 ± 0.44	4.96 ± 0.87	4.97 ± 0.77
**Data represents mean ± SD. No values were statistically significant with a p-value < 0.05.**				

Supplemental Table 1: Alpha Diversity Measures

Supplemental Figure 1. Blood metabolic parameters were measured using the Metlyte +CRP panel on a Piccolo Xpress. Data represent individual changes from baseline values for placebo and DE111treatment groups and horizontal lines represent means of each dataset. No values were statistically significant with a p-value <0.05. Abbreviations: Blood Urea Nitrogen (BUN); Creatinine (CRE); Creatinine Kinase (CK); Sodium (NA); Potassium (K), Total Carbon Dioxide (tCO2).

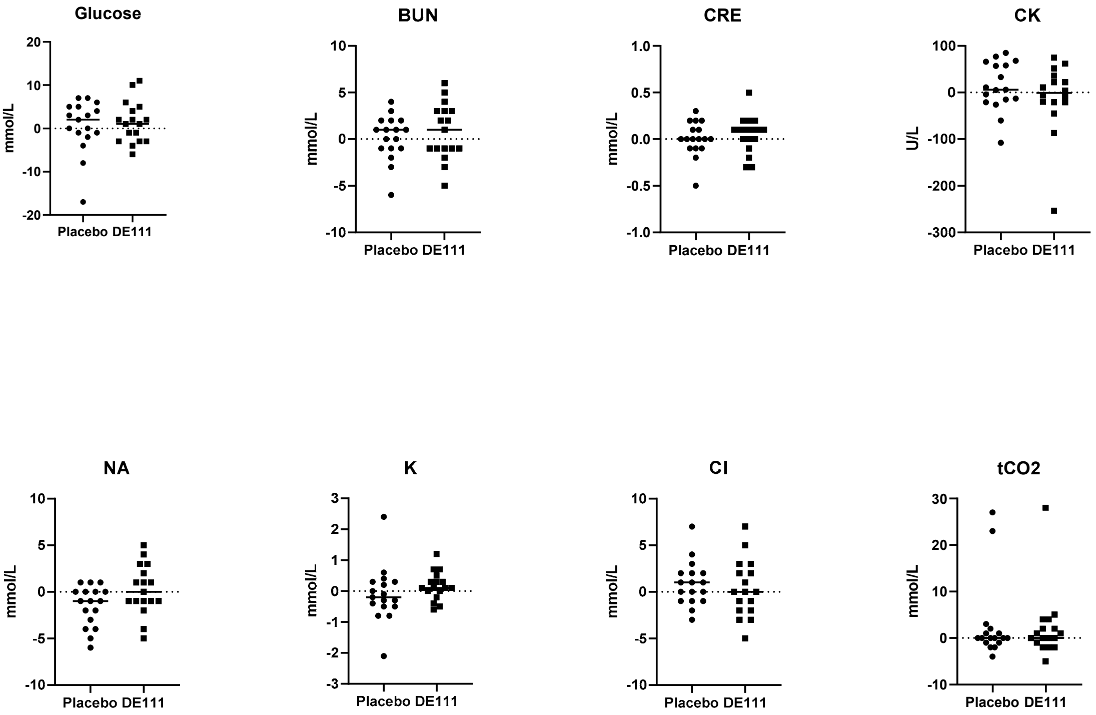

Supplemental Figure 2. Percent proliferating cells determined using proliferation marker CSFE. Data represent proliferating cells/total cells x 100 for a given cell type. The data represent mean ± SEM. There were no significant differences (p<0.05) either within (time) or between (treatment) treatment groups.

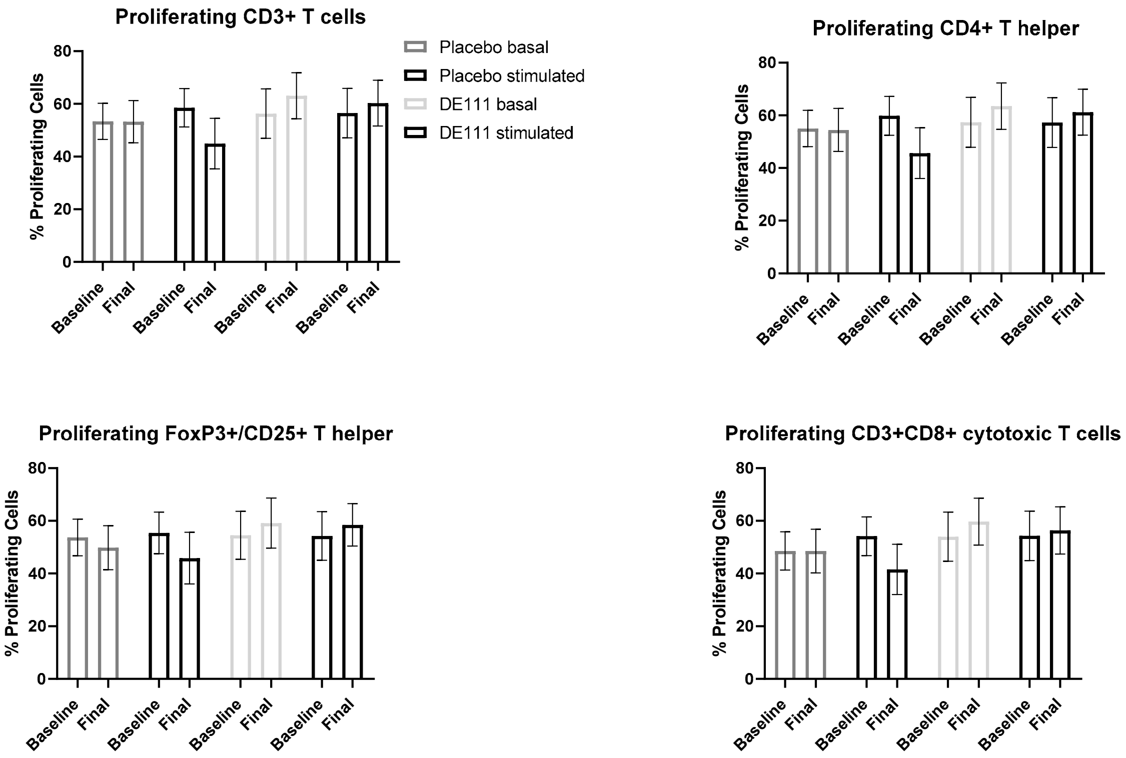

Supplemental Figure 3. Circulating Il-6 and zonulin as well as fecal sIgA were measured to assess systemic and mucosal inflammation as well as gut barrier function. There were no significant differences within (time) or between (treatment) treatment groups using $p<0.05$. Data represent means ±SEM.

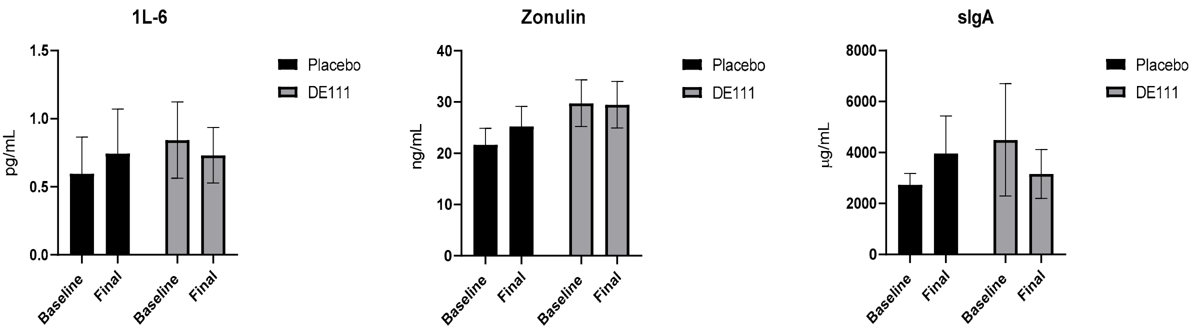

Supplement: Supplementary file 1 [file ijms-22-02453-s001.pdf]
